# A disproportionality analysis of adverse events associated to pertuzumab in the FDA Adverse Event Reporting System (FAERS)

**DOI:** 10.1186/s40360-023-00702-w

**Published:** 2023-11-13

**Authors:** Shu-peng Zou, Hai-yun Yang, Meng-ling Ouyang, Qian Cheng, Xuan Shi, Ming-hui Sun

**Affiliations:** 1 Department of Pharmacy, Tongji Hospital, Tongji Medical College, Huazhong University of Science and Technology, No.1095 Jiefang Avenue, Wuhan, Hubei Province 430000 China; 2 School of Pharmacy, Lanzhou University, Lanzhou, Gansu Province 730000 China

**Keywords:** Pertuzumab, FAERS, Pharmacovigilance, Adverse events

## Abstract

**Background:**

Pertuzumab is widely used for the treatment of HER2 + breast cancer. But its safety in the real world should be continuously monitored. So, we evaluated the safety of pertuzumab by pharmacovigilance analyze based on related adverse events (AEs) from the FDA Adverse Event Reporting System (FAERS) and find whether potential or uncertain adverse events were present.

**Methods:**

In disproportionality analysis, four algorithms were employed to detect the signals of pertuzumab from the FAERS between 2012 and 2022. In addition, we also used MYSQL 8.0, Navicat Premium 15, and Microsoft EXCEL 2019 to analyze the potential and high-ROR (reporting odds ratio) signals of pertuzumab. We also collected the onset times of pertuzumab-associated AEs.

**Results:**

From January 2012 to December 2022, there are 39,190,598 AEs reported from the FAERS database, of which 14,707 AEs listed pertuzumab as the ‘primary suspected (PS)’ drug. A total of 115 (46 potential) significant disproportionality preferred terms (PTs) conforming to the four algorithms were retained. Finally, we detected that the pertuzumab-induced AEs occurred in 12 organ systems. For pertuzumab, unexpected and significant PTs of AEs were found, including but not limited to below PTs: haematotoxicity, cardiotoxicity, cardiomyopathy, mitral valve incompetence, tachycardia, intestinal perforation, hemorrhoids, erysipelas, dehydration, pneumonitis, skin toxicity, onychomadesis, cyanosis, and circulatory collapse. We found there were 9 strong signals (5 potential safety signals) and 68 medium intensity signals (21 potential safety signals) according to IC_025_ (information component). The potential strong signals (IC_025_ > 3.0) were myelosuppression, cardiotoxicity, cardiac dysfunction, ejection fraction decreased, interstitial lung disease, and onychomadesis. Excluding unreported or unreasonable onset time reports, a total of 2016 AEs reported onset time and the median onset time was 117 days (4, 96), as median (Q1, Q3). Notably, most of the all AEs (*n* = 1133, 56%) and cardiac-related events (*n* = 405, 53%) all occurred within one month after pertuzumab therapy.

**Conclusion:**

Analysis of FAERS data identified pertuzumab-associated AEs, and our findings supported continuous clinical monitoring, pharmacovigilance, and further studies of pertuzumab. A significant association was detected between pertuzumab and some potential adverse events which should be regarded with some care. We have to pay attention to the first month after pertuzumab therapy and prepare emergency measures, especially for the elderly and patients with cardiovascular diseases.

**Supplementary Information:**

The online version contains supplementary material available at 10.1186/s40360-023-00702-w.

## Background

As early as 2020, breast cancer (BC) has become the commonly cancer in female and 10–15% of women will be possibly diagnosed with breast cancer at some time of their lifetime [1–3]. Noteworthily, 15-20% of patients with breast cancer overexpress the human epidermal growth factor receptor (EGFR) 2, also known as HER2, ERBB2 or CD340 [4–6]. Because of expensive treatments, a poor prognosis, and high recurrence rates, its burden has been rising over the past decades [5]. HER2 is different from other identified extracellular ligands (such as HER1, HER3, and HER4), because HER2 does not directly bind to any ligands [7]. It leads to that high expression of HER2 on cell surface has been used as an ideal target by different mechanisms and anti-HER2-directed agents have been developed in succession, such as trastuzumab, pertuzumab, afatinib, dacomitinib, margetuximab and antibody-drug conjugates (ADCs) [8–10]. However, the combination of trastuzumab, pertuzumab and taxane remains the preferred first-line therapy for HER2-positive metastatic breast cancer (MBC) in different guidelines [2, 4].

According gene expression patterns of previous studies, breast cancer is dived into five subtypes, known as the luminal A, luminal B, basal-like, HER2 overexpression, and normal breast-like subtype [11, 12]. The HER2 gene, as an actionable actuator, has advanced the development of HER-targeting monoclonal antibodies (mAbs) such as trastuzumab and pertuzumab, which resulted in the ameliorative survival time and quality of life [12–14]. HER2 mediates multiple signaling pathways in breast cancer and activates downstream signaling pathways to control cell growth, proliferation, differentiation, apoptosis, and metastasis [3]. Despite many conspicuous mAbs therapies developed, 15–25% of patients will still relapse in the early stage of BC and this causes treatments very difficult [15, 16].

In adverse events (AEs) of pertuzumab from the FAERS, females were accounted for a larger proportion than males which accord with the epidemiology of breast cancer. With the passage of time, FDA-approved HER2-targeted therapies has been more and more, including monoclonal antibodies (e.g., trastuzumab and pertuzumab), antibody-drug conjugates (e.g., trastuzumab emtansine), and small-molecule HER1/2 TKIs (e.g., lapatinib, neratinib, and tucatinib) [3, 17]. Additional anti-HER2 targeted treatment clinical studies are now being conducted. This significantly lessens the strain on breast cancer sufferers.

Although trastuzumab, the first HER2-targeted medication authorized by the FDA, significantly improved PFS (progression-free survival) in patients with HER2-positive breast cancer, approximately 25% patients will still relapse within the first decade after trastuzumab treatment [18]. Another randomized trial brought hope showing the addition of trastuzumab to chemotherapy could improve the survival outcomes of patients with metastatic HER2 + BC and reduce the recurrence [19]. Subsequently, a long-term follow-up study indicated that compared with two years of trastuzumab, one year of adjuvant trastuzumab after chemotherapy for HER2-positive BC improved long-term PFS significantly [15].

A report of drug reactions with HER2-Positive BC from the Italian pharmacovigilance database, showed serious AEs reports of anti-HER2 therapy mainly involved the following: thrombocytopenia, diarrhea, asthenia, cardiac failure, vomiting, hypersensitivity, ejection fraction decreased and stomatitis [20]. For the safety of trastuzumab emtansine (T-DM1) and trastuzumab deruxtecan (T-DXd), a pharmacovigilance study based on the FAERS database reported that T-DXd was more likely to induce ILD (interstitial lung disease)/pneumonia and myelosuppression than T-DM1, whereas T-DM1 had higher risk of hepatotoxicity, cardiotoxicity, and thrombocytopenia than T-DXd [21]. Compared anti-HER2 monotherapies and combination regimens, an analysis based on the FAERS showed that trastuzumab and pertuzumab/T-DM1 had higher odds of heart failure reporting than other anti-HER2 therapies [22].

Pertuzumab binds to the extracellular domain II (subdomain II) of HER2 receptor, different from the other domain of trastuzumab (subdomain IV) [23]. So, the combination of trastuzumab and pertuzumab can multiply inhibit Her2 signaling and interact with immune effector cell Fc receptors to cause antibody dependent cellular cytotoxicity (ADCC) [24]. The previous CLEOPATRA trial demonstrated that pertuzumab, combined with trastuzumab and docetaxel chemotherapy, significantly improved PFS and overall survival (OS) compared with trastuzumab and docetaxel alone [25]. Although the development of antibody drug conjugates (ADCs), ASCO (American Society of Clinical Oncology) guideline indicated that the dual HER2-targeted treatment including pertuzumab and trastuzumab, additional chemotherapy (taxane) remained the first-line standard therapy for patients with HER2 + BC [17].

Pertuzumab-related AEs (≥ Grade 3, severe adverse events, MedDRA) were reported as cardiotoxicity, diarrhea, neutropenia and left ventricular systolic dysfunction [26]. But in CLEOPATRA trial (NCT00567190), no heart failure, symptomatic left ventricular systolic dysfunction, or left ventricular ejection fraction decline (< 40%) were reported [26]. While, safety was similar in many clinical trials with no potential safety signals [26–31]. In addition, the FAERS, as a spontaneous report system, could display more information than clinical trials, like non-selective sampling.

Compared with trastuzumab and chemotherapy, additional pertuzumab for treatment of metastatic breast cancer can shorten the median OS and improved progression-free survival (PFS) obviously [30, 32]. Furthermore, there were also no significant differences for heart failure–related and febrile neutropenia–related adverse events in the first-line standard therapy [32, 33]. In a random phase III study of pertuzumab, trastuzumab, and docetaxel, no heart failure cases or symptomatic LVEF (Left ventricular ejection fraction) declines were reported [34]. Similarly, in the APHINITY study of cardiac safety of pertuzumab plus trastuzumab, the dual blockade did not increase the risk of cardiac events (CEs) compared with trastuzumab alone and the anthracycline-based chemotherapy increased the risk of a CE [35]. But in FDA label, pertuzumab can result in subclinical and clinical cardiac failure manifesting as decreased LVEF and congestive heart failure (CHF) [36]. So, there were not enough evidences to confirm the cardiotoxicity and if there were potential adverse events occurred. In this article, we use the disproportionality analysis to find the potential safety signals of pertuzumab, comprehensively.

## Methods

### Data sources

We collected the data of the drug (pertuzumab, Perjeta) from the FAERS from January 2012 to December 2022. The FAERS, as a spontaneous report system, its adverse events including update information were submitted by medical personnel, consumers, manufacturers and others. In addition, we removed duplicate individual safety reports with the same identifier number from demographic (DEMO) file and performed the interlink of reaction (REAC) file with the MedDRA by using PTs [37].

### Data mining

We applied disproportionality analysis to evaluate the safety signals for patients with pertuzumab and its types in medical subject headings [MeSH]. We chose “pertuzumab” as the target drugs, and primary suspect drugs as the drug role code in the dataset [38]. Otherwise, we used MySQL (version 8.0) and Navicat (Navicat Premium 16.0) to establish the dataset from FAERS. The outcomes of the dataset were standardized vocabularies with drug names corresponding to the preferred terms (PTs) from Medical Dictionary for Regulatory Activities (MedDRA®) (version 24.0) and also used Excel software to compute drug-reaction signals statistically [39].

### Statistical analysis

A disproportionality analysis is commonly used to analyze post-marketing surveillance databases to explore potential associations between drugs and adverse events [40, 41]. We completed the normal operation including disposal, cleaning, collecting, and calculating the signals of clinical characteristics from the dataset by MySQL (version 8.0), Microsoft EXCEL, and Navicat (Navicat Premium 16.0) [42]. Based on a fourfold table, the reporting odds ratio (ROR), the proportional reporting ratio (PRR), the Bayesian confidence propagation neural network (BCPNN), and the multi-item gamma Poisson shrinker (MGPS) were applied to detect an association between various pertuzumab regimens and adverse events in accordance with the disproportionality analysis [40, 43–48]. This method compares the proportion of a certain event of the target drug in the ADE spontaneous reporting system with the proportion of the target event of all other drugs (background data) [49]. The equations and criteria for the above four algorithms are shown in Supplementary Table S1. We will investigate the statistical association between this targeted drug and the event to detect potential AE signals. A positive signal was accord with each criterion of four algorithms. The time-to-onset of adverse events used the formula as follows:

$$Time-to-onset=Event\;onset\;date\;(EVENT\_DT)-Therapy\;start\;date\;(START\_DT)$$,

after we removed the reports with obvious errors such as illogical dates, such as missing data and the negative number of time-to-onset. The onset time of AEs induced by pertuzumab was calculated and shown as the percentage, expressed as “%” [50].

## Results

### Descriptive analysis

**Fig. 1:**
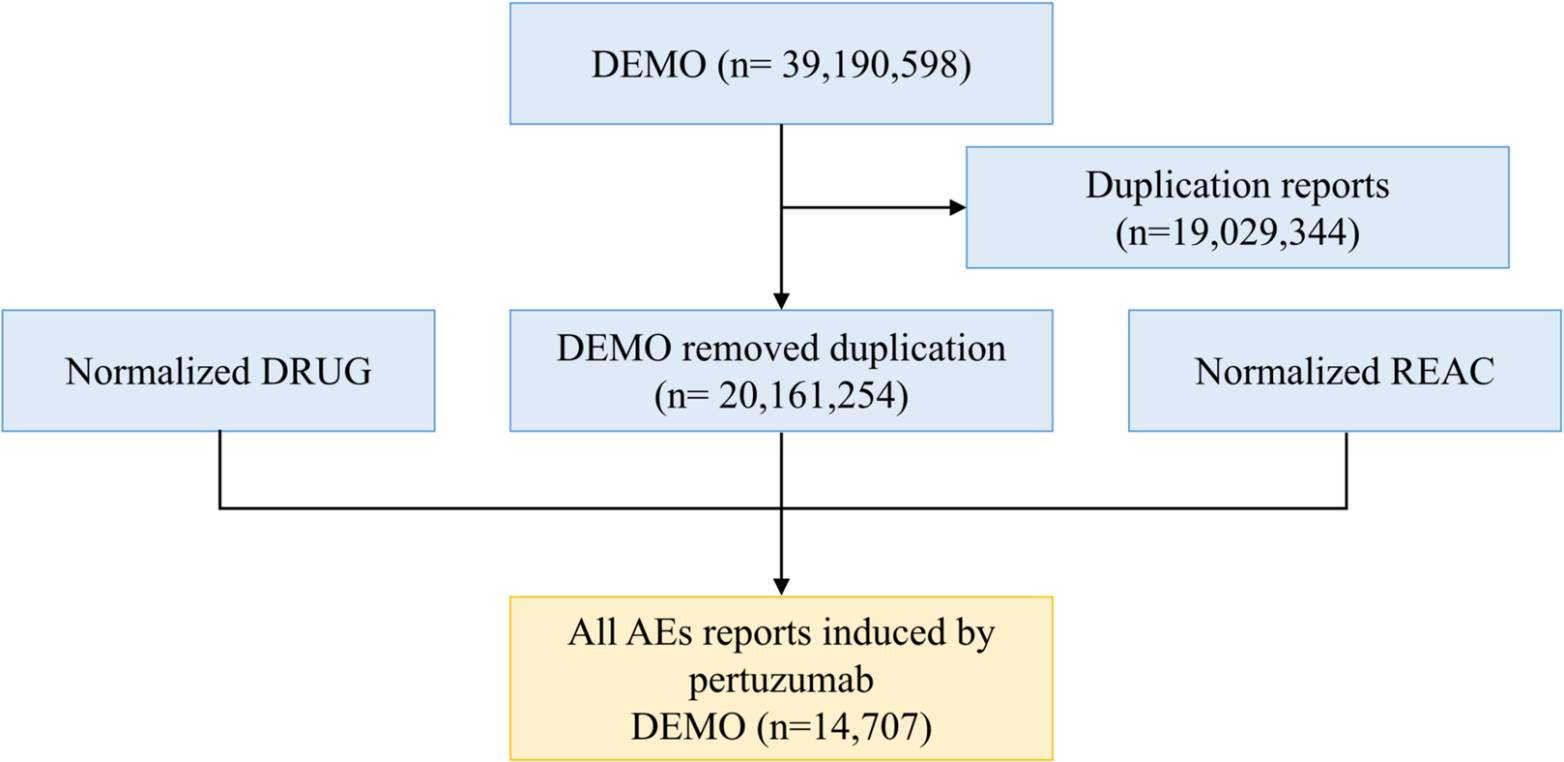
The process of searching pertuzumab-associated adverse events from food and drug administration adverse event reporting database (FAERS)

From January 2012 to December 2022, there are 39,190,598 AEs reported in the FAERS database in Fig. 1. Of which, 14,707 AEs excluded repetitive and missing data were found to be related to pertuzumab. The clinical characteristics of pertuzumab were showed in Table 1. Among all AEs, females (12,903, 87.7%) were accounted for a larger proportion than males (246, 1.7%). As the Table 1 shows, the most reported indication was breast cancer (5682, 38.6%), followed by HER-2 positive breast cancer (3238, 22.0%) and breast cancer metastatic (2159, 14.9%). In our collected data, the median age of patients was 56.4 (48, 65), as median (Q1, Q3). In outcomes of pertuzumab treatments, in addition to other serious medical events (2247, 15.3%), hospitalization-initial or prolonged (1787, 12.2%) was the most frequently reported serious outcome. Most of AEs were reported from United States (4696, 31.9%), followed by Canada (1574, 10.7%), Germany (1360, 9.2%), China (870, 5.9%), and Great Britain (839, 5.7%). Physician (7408, 50.4%) reported the most, followed by consumers (2583, 17.6%). In terms of reporting years, the most reported year was 2022 (2673, 18.2%), followed by 2021 (2615, 17.8%), 2020 (2107, 14.3%), and 2019 (1650, 11.2%), respectively.

**Table 1:** Characteristics of reports from the FAERS database (January 2012 to December 2022)

	Pertuzumab, *N* (%)
**Gender**	
Female	12,903 (87.7)
Male	246 (1.7)
Unknown	1558
**Age**	
Median (Q1,Q3)	56.4 (48,65)
**Country of the report**	
United States	4696 (31.9)
Canada	1574 (10.7)
Germany	1360 (9.2)
China	870 (5.9)
Great Britain	839 (5.7)
**Reporter**	
Physician	7408 (50.4)
Consumer	2583 (17.6)
Other health-professional	1703 (11.6)
Pharmacist	1293 (8.8)
**Indications**	
Breast cancer	5682 (38.6)
HER-2 positive breast cancer	3238 (22.0)
Breast cancer metastatic	2159 (14.9)
Invasive ductal breast carcinoma	226 (1.5)
Premedication	216 (1.5)
**Outcome**	
Other Serious (Improtant Medical Event)	2247 (15.3)
Hospitalization-Initial or Prolonged	1787 (12.2)
Death	630 (4.3)
Life-Threatening	250 (1.7)
Disability	70 (0.5)
**Reporting year (5 years)**	
2022	2673
2021	2615
2020	2107
2019	1650
2018	1537

*N* The number of reports, *Q *Quarter, *HER-2 *Human epidermal growth factor receptor

### Signal of system organ class

Based on the original data, all cases with missing doses, frequencies, or indications were excluded. The signal strength of AEs of pertuzumab at the System Organ Class (SOC) level are described in Table 2. Finally, we detected that pertuzumab- induced AEs occurred in targeting 12 organ systems. The significant SOCs of pertuzumab that at least one of the four indices accorded with the criteria were blood and lymphatic system disorders (SOC: 10005329, 848), cardiac disorders (SOC: 10007541, 645), gastrointestinal disorders (SOC: 10017947, 2104), respiratory, thoracic and mediastinal disorders (SOC: 10038738, 1088), skin and subcutaneous tissue disorders (SOC: 10040785, 1132), hepatobiliary disorders (SOC: 10019805, 171), vascular disorders (SOC: 10047065, 414), investigations (SOC: 10022891, 1067) and so on.

**Table 2:** Signal strength of adverse events of pertuzumab at the system organ class (SOC) level in FAERS database

System Organ Class (SOC)	Cases Reporting SOC	ROR	PRR (χ^2^)	IC (IC_025_)	EBGM (EBGM 05)
Blood and lymphatic system disorders	848	3.73 (3.48-4.00) ^a^	3.57 (68673.87) ^a^	1.84 (1.71) ^a^	3.57 (3.37) ^a^
Cardiac disorders	645	1.91 (1.76–2.06) ^a^	1.87 (16674.70)	0.90 (0.83) ^a^	1.87 (1.75)
Gastrointestinal disorders	2104	1.80 (1.72–1.89) ^a^	1.69 (145491.33)	0.75 (0.72) ^a^	1.69 (1.62)
Respiratory, thoracic and mediastinal disorders	1088	1.63 (1.53–1.73) ^a^	1.58 (30352.29)	0.66 (0.62) ^a^	1.58 (1.50)
Skin and subcutaneous tissue disorders	1132	1.42 (1.34–1.51) ^a^	1.39 (19323.01)	0.47 (0.45) ^a^	1.39 (1.32)
Hepatobiliary disorders	171	1.42 (1.22–1.65) ^a^	1.41 (457.89)	0.50 (0.43) ^a^	1.41 (1.25)
Vascular disorders	414	1.38 (1.25–1.52) ^a^	1.37 (2273.62)	0.45 (0.41) ^a^	1.37 (1.26)
Metabolism and nutrition disorders	393	1.29 (1.17–1.43) ^a^	1.28 (1378.17)	0.36 (0.32) ^a^	1.28 (1.18)
Investigations	1067	1.25 (1.18–1.34) ^a^	1.24 (7998.15)	0.31 (0.29) ^a^	1.24 (1.17)
Infections and infestations	961	1.25 (1.17–1.34) ^a^	1.24 (6480.47)	0.31 (0.29) ^a^	1.24 (1.17)
Immune system disorders	190	1.12 (0.97–1.29)	1.12 (75.71)	0.16 (0.14) ^a^	1.12 (0.99)
Neoplasms benign, malignant and unspecified (incl cysts and polyps)	464	1.06 (0.97–1.17)	1.06 (131.92)	0.09 (0.08) ^a^	1.06 (0.98)

*ROR* Reporting odds ratio, *CI *Confidence interval, *PRR* Proportional reporting ratio, *χ* Chi-squared, *IC* Information component, *IC*_*025*_ The lower limit of 95% CI of the IC, *EBGM* Empirical Bayesian geometric mean, *EBGM*_*05*_ The lower limit of 95% CI of EBGM

^a^Indicates statistically significant signals in algorithm

### Signal of preferred terms

We detected suspicious signals of pertuzumab by four pharmacovigilance algorithms (ROR, PRR, BCPNN, and MGPS) and showed the results in Table 3. First, we evaluated preferred terms (PT) levels from MedDRA® to describe the toxicity spectrum of pertuzumab. In Supplementary Table S2, we further examined all PT signals and a total of 116 (46 potential PTs) PTs significant disproportionality PTs conformed to the four algorithms simultaneously. Blood system events, cardiac disorders events, gastrointestinal disorders events, and respiratory system events that are included in the label are usually reported in patients with pertuzumab.

**Table 3:** New signal strength of partial preferred terms (PTs) of pertuzumab from FAERS database

System Organ Class (SOC)	Preferred Terms (PTs)	Cases Reporting PTs	ROR	PRR (χ^2^)	IC (IC_025_)	EBGM (EBGM_05_)
Blood and lymphatic system disorders	Myelosuppression*	305	86.09 (76.72–96.59)	84.32 (16461.01)	6.35 (5.66)^a^	81.77 (74.26)
	Haematotoxicity*	8	3.89 (1.95–7.79)	3.89 (6.40)	1.96 (0.98)	3.89 (2.18)
Cardiac disorders	Cardiotoxicity*	70	35.04 (27.66–44.38)	34.88 (837.72)	5.11 (4.03) ^a^	34.44 (28.26)
	Cardiac dysfunction*	27	34.95 (23.90-51.11)	34.89 (124.62)	5.11 (3.49) ^a^	34.45 (25.07)
	Left ventricular dysfunction	27	17.27 (11.83–25.23)	17.24 (117.22)	4.10 (2.81)^b^	17.14 (12.48)
	Ventricular hypokinesia	8	12.39 (6.18–24.81)	12.38 (9.80)	3.62 (1.81) ^b^	12.33 (6.89)
	Cardiomyopathy*	22	6.98 (4.59–10.60)	6.97 (64.34)	2.80 (1.84) ^b^	6.95 (4.90)
	Mitral valve incompetence*	9	4.38 (2.28–8.42)	4.38 (8.74)	2.13 (1.11)	4.37 (2.53)
	Sinus tachycardia*	10	3.44 (1.85–6.40)	3.44 (9.11)	1.78 (0.96)	3.43 (2.04)
	Tachycardia*	65	3.33 (2.61–4.25)	3.32 (374.13)	1.73 (1.36)	3.32 (2.70)
Gastrointestinal disorders	Enterocolitis infectious*	6	29.56 (13.22–66.11)	29.55 (6.09)	4.87 (2.18) ^b^	29.24 (14.91)
	Enterocolitis*	11	8.58 (4.75–15.51)	8.58 (17.11)	3.10 (1.71) ^b^	8.55 (5.21)
	Intestinal perforation*	10	3.87 (2.08–7.20)	3.87 (9.97)	1.95 (1.05)	3.87 (2.30)
	Colitis*	27	3.15 (2.16–4.60)	3.15 (61.50)	1.65 (1.13)	3.14 (2.29)
	Haemorrhoids*	15	3.09 (1.86–5.13)	3.09 (18.64)	1.63 (0.98)	3.09 (2.02)
General disorders and administration site conditions	Device related thrombosis*	5	19.48 (8.08–46.97)	19.48 (4.08)	4.27 (1.77) ^b^	19.34 (9.26)
	Mucosal disorder*	5	19.26 (7.99–46.42)	19.25 (4.07)	4.26 (1.77) ^b^	19.12 (9.16)
	Hyperpyrexia	14	16.56 (9.79–28.01)	16.54 (31.35)	4.04 (2.39) ^b^	16.45 (10.59)
	Performance status decreased*	11	10.92 (6.04–19.75)	10.92 (18.09)	3.44 (1.90) ^b^	10.88 (6.63)
	Temperature intolerance*	12	4.91 (2.79–8.65)	4.91 (16.54)	2.29 (1.30)	4.90 (3.05)
Hepatobiliary disorders	Hepatic lesion*	6	5.99 (2.69–13.34)	5.98 (4.52)	2.58 (1.16)	5.97 (3.05)
Immune system disorders	Neutropenic sepsis	20	11.75 (7.57–18.23)	11.73 (60.65)	3.55 (2.29) ^b^	11.69 (8.09)
	Erysipelas*	13	10.51 (6.10-18.13)	10.51 (25.07)	3.39 (1.96) ^b^	10.47 (6.64)
	Paronychia	10	9.81 (5.27–18.26)	9.80 (14.61)	3.29 (1.77) ^b^	9.77 (5.81)
	Subcutaneous abscess*	9	7.52 (3.91–14.46)	7.51 (11.03)	2.91 (1.51) ^b^	7.49 (4.33)
	Rash pustular*	11	6.29 (3.48–11.37)	6.29 (15.50)	2.65 (1.47)	6.27 (3.82)
	Anaphylactic shock	28	5.40 (3.72–7.82)	5.39 (94.26)	2.43 (1.68) ^b^	5.38 (3.94)
	Cellulitis*	48	3.83 (2.89–5.09)	3.82 (227.82)	1.93 (1.46)	3.82 (3.01)
	Gastroenteritis*	12	3.67 (2.08–6.46)	3.67 (13.80)	1.87 (1.06)	3.66 (2.28)
Injury, poisoning and procedural complications	Radiation necrosis*	5	47.26 (19.52-114.45)	47.24 (4.34)	5.54 (2.29) ^b^	46.44 (22.16)
	Radiation skin injury	6	22.37 (10.01–49.97)	22.36 (5.95)	4.47 (2.00) ^b^	22.18 (11.32)
Investigations	Carbohydrate antigen 15 − 3 increased*	6	36.77 (16.42–82.31)	36.75 (6.17)	5.18 (2.31) ^b^	36.27 (18.48)
	Mean platelet volume decreased*	7	36.31 (17.22–76.55)	36.29 (8.40)	5.16 (2.45) ^b^	35.81 (19.19)
	Ejection fraction decreased	109	31.58 (26.13–38.17)	31.35 (2017.95)	4.95 (4.10) ^a^	31.00 (26.45)
	Echocardiogram abnormal*	5	18.64 (7.73–44.93)	18.63 (4.06)	4.21 (1.75) ^b^	18.51 (8.87)
	Red cell increased	12	11.85 (6.72–20.89)	11.84 (21.87)	3.56 (2.02) ^b^	11.79 (7.33)
	Pulse absent*	8	9.02 (4.51–18.06)	9.02 (9.17)	3.17 (1.58) ^b^	8.99 (5.03)
	Blood magnesium decreased*	13	6.42 (3.72–11.06)	6.41 (21.82)	2.68 (1.55) ^b^	6.40 (4.06)
	SARS-CoV-2 test positive*	15	3.98 (2.40–6.60)	3.97 (22.83)	1.99 (1.20)	3.97 (2.60)
Metabolism and nutrition disorders	Dehydration*	127	4.21 (3.54–5.02)	4.19 (1696.31)	2.06 (1.73) ^b^	4.18 (3.61)
	Hypomagnesaemia*	12	3.93 (2.23–6.93)	3.93 (14.51)	1.97 (1.12)	3.93 (2.44)
Nervous system disorders	Polyneuropathy*	24	9.06 (6.07–13.53)	9.05 (82.58)	3.17 (2.12)	9.02 (6.45)
	Intracranial pressure increased*	6	4.87 (2.18–10.84)	4.86 (4.12)	2.28 (1.02)	4.86 (2.48)
Respiratory, thoracic and mediastinal disorders	Nasal ulcer*	6	14.95 (6.70-33.35)	14.94 (5.68)	3.89 (1.75) ^b^	14.86 (7.59)
	Interstitial lung disease*	136	12.78 (10.79–15.13)	12.67 (2844.95)	3.66 (3.09) ^a^	12.61 (10.95)
	Pneumonitis*	52	8.54 (6.50-11.22)	8.52 (381.76)	3.09 (2.35) ^b^	8.49 (6.76)
	Lung infiltration*	13	7.85 (4.55–13.54)	7.85 (23.31)	2.97 (1.72) ^b^	7.83 (4.96)
	Bronchospasm*	23	7.36 (4.88–11.08)	7.35 (71.54)	2.87 (1.91) ^b^	7.33 (5.20)
	Pulmonary hypertension*	16	3.23 (1.98–5.28)	3.23 (22.11)	1.69 (1.03)	3.23 (2.14)
Skin and subcutaneous tissue disorders	Onychomadesis*	21	26.77 (17.41–41.16)	26.74 (74.04)	4.73 (3.07) ^a^	26.48 (18.48)
	Dermatitis acneiform*	20	14.86 (9.57–23.06)	14.84 (63.04)	3.88 (2.50) ^b^	14.76 (10.22)
	Nail discolouration*	10	10.13 (5.44–18.85)	10.12 (14.72)	3.33 (1.79)	10.09 (6.00)
Vascular disorders	Cyanosis*	15	4.85 (2.92–8.06)	4.85 (25.70)	2.28 (1.37)	4.84 (3.17)
	Lymphoedema*	14	8.39 (4.96–14.18)	8.38 (27.55)	3.06 (1.81) ^b^	8.36 (5.39)
	Circulatory collapse*	13	3.49 (2.02–6.01)	3.48 (15.57)	1.80 (1.04)	3.48 (2.21)

*ROR* Reporting odds ratio, *CI *Confidence interval, *PRR *Proportional reporting ratio, *χ *Chi-squared, *IC *Information component, *IC025 *The lower limit of 95% CI of the IC, *EBGM *Empirical Bayesian geometric mean, *EBGM05* The lower limit of 95% CI of EBGM

*Emerging findings of pertuzumab associated PTs from FAERS database; a: IC025 > 3.0, it indicates a strong signal; b: 1.5 < IC025 ≤ 3.0, it indicates a medium intensity signal

In Table 3, we enumerated the potential and suspicious PTs signals of pertuzumab from the FAERS database. In the analysis of pertuzumab, unexpected significant AEs were found in Table 3, including but not limited to below PTs: myelosuppression (ROR 86.09; PT 10028584), haematotoxicity (ROR 3.89; PT 10061188), cardiotoxicity (ROR 35.04; PT 10048610), cardiomyopathy (ROR 6.98; PT 10007636), mitral valve incompetence (ROR 4.38; PT 10027727), tachycardia (ROR 3.33; PT 10043071), intestinal perforation (ROR 3.87; PT 10022694), hemorrhoids (ROR 3.09; PT 10019022), erysipelas (ROR 10.51; PT 10015145), carbohydrate antigen 15 − 3 increased (ROR 36.77; PT 10051415), dehydration (ROR 4.21; PT 10012174), pneumonitis (ROR 8.54; PT 10035742), onychomadesis (ROR 26.77; PT 10049274), cyanosis (ROR 4.85; PT 10011703), and circulatory collapse (ROR 3.49; PT: 10009192). If IC_025_ (the lower limit of 95% CI of the BCPNN) > 3.0, it indicated a strong signal and if 1.5 < IC025 ≤ 3.0, it indicated a medium intensity signal [51]. So, we found there were 9 strong signals (5 potential safety signals) and 69 medium intensity signals (22 potential safety signals). The potential strong signals (IC_025_ > 3.0) were myelosuppression, cardiotoxicity, cardiac dysfunction, ejection fraction decreased, interstitial lung disease, and onychomadesis.

### Onset time of pertuzumab-related AEs

**Fig. 2:**
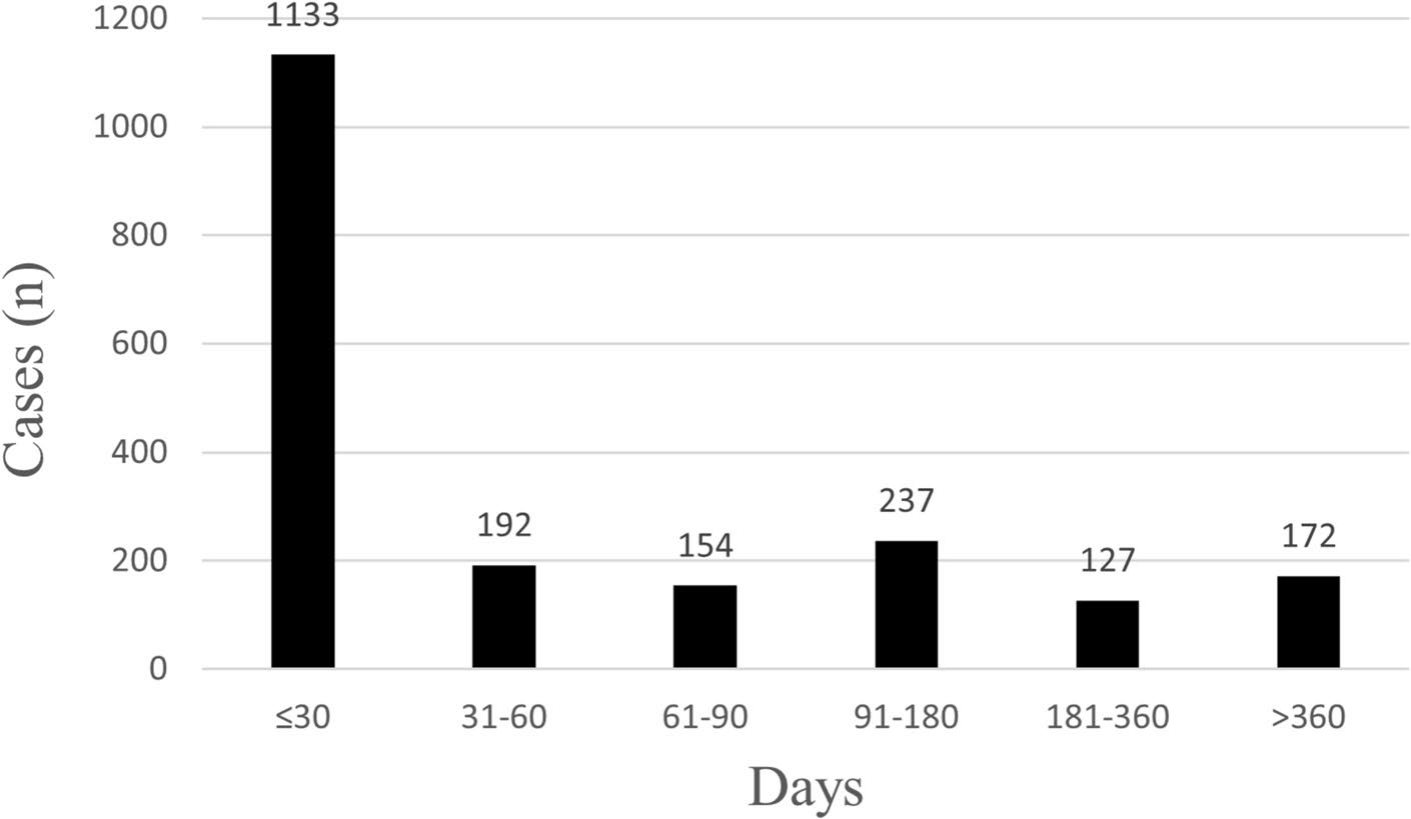
Time to onset of pertuzumab-related AEs. AEs: Adverse Events. (The unit: days)

We collected the onset times of pertuzumab-associated AEs from the FAERS database and showed the results in Fig. 2. Excluding unreported or unreasonable onset time reports such that event onset date was before therapy start date, a total of 2016 AEs reported onset time and the median onset time was 117 days (4, 96), as median (Q1, Q3). In Fig. 2, most of the AEs occurred within 1 month (*n* = 1133, 56%) after pertuzumab therapy. Notably, we also found that in 764 cardiac-related events, most of the AEs also occurred within 1 month (*n* = 405, 53%) after pertuzumab therapy.

## Discussion

To the best of our knowledge, this large real-world comparison of pertuzumab used the FAERS data firstly. Our study showed that the most commonly reported and fire-potential safety signals were found at PT levels. In the SOC level, blood and lymphatic system disorders were the most commonly reported and signficant signals. In contrast, signficant disproportionality of AEs in cardiac disorders, gastrointestinal disorders, respiratory disorders, skin disorders, hepatobiliary disorders, vascular disorders, and investigations were less common.

HER2 + inhibitors such as trastuzumab and pertuzumab are not only widely used in breast cancer, but also other HER-positive diseases such as colorectal cancer, metastatic gastric cancer, biliary tract cancer, and leptomeningeal disease due to the susceptibility gene and PI3K-signaling pathway [52–54]. Physicians may need to exercise additional caution on blood, cardiac, and gastrointestinal system disorders while using pertuzumab. In an analysis of anti-HER2 therapy from the Italian pharmacovigilance database, cardiac failure, vascular disorder and infusion-related reactions with hypersensitivity were more frequent in the treatment of pertuzumab and trastuzumab [20]. According to the BC Cancer Agency (BCCA) drug assessment report, 13.0–40.0% of patients treated with pertuzumab have been observed infusion-related reactions, including symptoms of asthenia, chills, fatigue, and hypersensitivity [20, 55].

Pertuzumab has been often used in conjunction with other HER2-targeted drugs and chemotherapy such as trastuzumab, trastuzumab emtansine (T-DM1), atezolizumab, docetaxel, and taxane, significantly improving PFS or delaying brain metastases in patients with BC [56]. But, some therapies express some more serious AEs, studies have shown that major severe AEs of T-DM1 + pertuzumab ± taxane included thrombocytopenia, neutropenia, fatigue, increased ALT, anemia and peripheral neuropathy [57]. However, the addition of pertuzumab to T-DM1 ± taxane only led to higher risks of diarrhea (especially grade ≥ 3 diarrhea), rash and vomiting, and decreased risks of thrombocytopenia [58]. In the real-world, we found that whether it was in combination or not, pertuzumab as the primary suspect drug was detected a total of 115 (46 in potential) significant PTs signals, such as haematotoxicity, cardiotoxicity, cardiomyopathy, mitral valve incompetence, tachycardia, intestinal perforation, hemorrhoids, erysipelas, carbohydrate antigen 15 − 3 increased, dehydration, pneumonitis, skin toxicity, onychomadesis, cyanosis, and circulatory collapse. We also found there were 9 strong signals (5 potential safety signals) and 68 medium intensity signals (21 potential safety signals) in the PTs level. The potential strong signals (IC_025_ > 3.0) were myelosuppression, cardiotoxicity, cardiac dysfunction, ejection fraction decreased, interstitial lung disease, and onychomadesis. Noteworthily, pertuzumab currently have not a ‘boxed warning’ for an obvious risk of myelosuppression in its FDA label.

For cardiovascular events, both HER2 receptors and their ligands are expressed in cardiac cells; inhibition of these pathways may affect the ability of the heart to withstand stress and thus impact cell survival [59]. In a pharmacovigilance analysis of anti-HER2 monotherapies, trastuzumab and pertuzumab/T-DM1 (12.04%) had a higher risk of heart failure than other anti-HER2 therapies (1–2%) [22]. So, pertuzumab may had a synergistic effect with trastuzumab on cardiac disorders in older patients. In a phase IIIb single‑arm safety study (NCT02402712), safety and efficacy with subcutaneous trastuzumab plus intravenous pertuzumab and docetaxel in BC are consistent with this intravenous combination, and the most common severe events were neutropenia, febrile neutropenia, and hypertension, with no cardiac deaths and stable LVEF [31].

Gastrointestinal events also were common in our analysis. We found some unexpected signals of pertuzumab including enterocolitis, intestinal perforation, colitis, and hemorrhoids, excepting other common events such as abdominal pain, nausea, and vomiting. The substantive enteropathy may be caused by the development of cancer diseases. Some studies have suggested that blocking EGFR can cause excess chloride secretion, resulting in impaired gut absorption and secretory diarrhea [60, 61]. Additionally, compared to trastuzumab plus chemotherapy, dual anti-HER2 blockade regimens revealed an increased probability of gastrointestinal reactions [62].

It’s worth noting that we found disproportionality reporting for skin and subcutaneous tissue disorders. In addition to breast and cardiac cells, HER2 is also expressed in keratinocytes [59]. Aside from reported skin- related AEs such as palmar-plantar erythrodysaesthesia syndrome, nail disorder, and skin reaction, we found potential strong signals of pertuzumab including nail discolouration (ROR = 10.1), ermatitis acneiform (ROR = 14.9) and onychomadesis (ROR = 26.8). Interestingly, onychomadesis was not found in any study of pertuzumab. Based on our disproportionality analysis, multiple organ systems were involved and the strongest signal was blood and lymphatic system (IC_025_ = 1.71, it indicated a medium intensity signal), follow by cardiac disorders, gastrointestinal disorders, respiratory, thoracic disorders, skin disorders, and hepatobiliary disorders.

In Supplementary Table S2, Metastases to central nervous system (ROR = 30.93) also showed a strong signal. According to statistics, approximately one-third of patients with HER2 + breast cancer will finally transfer to CNS (central nervous system) [63]. Despite this high incidence of CNS metastases, compared to other subtypes, patients with HER2-positive MBC achieve a better survival rate because of better systemic and cranial disease control provided by anti-HER2 agents [64–66]. Additionally, the phase I/II study showed that the outcomes of HER-positive leptomeningeal disease patients treated with intrathecal trastuzumab remained safe and well-tolerated [64]. To symptomatic brain metastases, the treatments including neurosurgery and/or radiotherapy are more depending on the number of metastases, performance status, and systemic disease control [4].

Results of this study indicated that the median onset time was 117 days (4, 96), as median (Q1, Q3) and most of the cases occurred within the first month (*n* = 1133, 56%) after pertuzumab. So, we have to pay attention to the first month after pertuzumab and take emergency measures, especially the elderly and patients with cardiovascular diseases.

In our study, we also have some limitations in the FAERS. Unfortunately, because the causal relationship for submitted reports and some information are lacking, such as underreporting, incomplete reporting, and false reporting, it is difficult to control confounding factors [67]. Second, because of the lack of total number of patients with pertuzumab treatment and the same event reported by two sources (physician and consumer) resulting in two IDs potentially, it is impossible to calculate relevant statistics such as the adverse reaction ratio. Third, because of a lack of information, the important risk factors of pertuzumab have become challenging to deal with. So, while data mining cannot compensate for the inherent limitations of self-reporting systems or replace expert review, it does have a place when large amounts of data are involved [68].

Although pertuzumab has some AEs, double anti-HER2 blockade associated with a taxane currently remains the best option in the first line. The standard of care was dual blockade with trastuzumab and pertuzumab as first-line, followed by TDM-1 as second-line [23]. With the development of related clinic trials, more effective and more safe therapeutic regimens will be brought to the patient with HER2+/- breast cancer.

## Conclusion

In conclusion, this is the first study to analyze pertuzumab from FAERS data comprehensively and systematically. It is very important for continuous monitoring of drug safety profiles in the real-world. For pertuzumab, unexpected and 46 potential significant PTs of AEs were found, such as cardiotoxicity, cardiomyopathy, mitral valve incompetence, cyanosis, lymphoedema, circulatory collapse, intestinal perforation, polyneuropathy, onychomadesis, interstitial lung diseases, tachycardia, and so on. In the onset times of all AEs and cardiovascular events, we must pay attention to the first month after pertuzumab.

### Supplementary Information

**Additional file 1.**

## Data Availability

The datasets generated and analyzed during the current study are available from the corresponding author on reasonable request.

## Abbreviations

ADCC: Antibody dependent cellular cytotoxicity
ADCs: Antibody-drug conjugates
AEs: Adverse events
ASCO: American Society of Clinical Oncology
BC: Breast cancer
BCPNN: Bayesian confidence propagation neural network
CEs: Cardiac events
CHF: Congestive heart failure
CNS: central nervous system
DEMO: Demographic
DFS: Disease-free survival
EGFR: Epidermal growth factor receptor
FDA: U.S. Food and Drug Administration
FAERS: FDA Adverse Event Reporting System
HER2: human epidermal growth factor receptor 2
IC: Information component
IC025: The lower limit of 95% confidence interval of the IC
ILD: Interstitial Lung Disease
LVEF: Left ventricular ejection fraction
mAbs: Monoclonal antibodies
MBC: Metastatic breast cancer
MedDRA: Medical Dictionary for Regulatory Activities
MGPS: Multi-item gamma Poisson shrinker
OS: Overall survival
PFS: Progression-free survival
PRR: Proportional reporting ratio
PS: Primary suspected
PTs: Preferred terms
REAC: Reaction
ROR: Reporting odds ratio
T-DM1: Trastuzumab emtansine
T-DXd: Trastuzumab deruxtecan
