# Supplementary material for: A disproportionality analysis of adverse events associated to pertuzumab in the FDA Adverse Event Reporting System (FAERS)
Source: BMC Pharmacol Toxicol. 2023 Nov 13;24:62. doi: 10.1186/s40360-023-00702-w (PMC10642055; doi:10.1186/s40360-023-00702-w)
Supplement: Supplementary file 1 — Additional file 1. [file 40360_2023_702_MOESM1_ESM.docx]

Supplementary Material

Post-marketing safety of Pertuzumab: an analysis of the FDA Adverse Event Reporting System (FAERS)

# Supplementary Tables

**Supplementary Table S1. Summary of major algorithms used for signal detection.**

| Algorithms | Equation | Criteria |
| --- | --- | --- |
| ROR | ROR=ad/bc | lower limit of 95% CI>1, N≥2 |
|  | 95%CI=e^ln(ROR)±1.96(1/a+1/b+1/c+1/d)^0.5^ |  |
| PRR | PRR=(a(c+d))/(c(a+b)) | PRR≥2, χ^2^≥4, N≥3 |
|  | χ^2^ = [(ad − bc)^2](a + b + c + d)/[(a + b)(c + d)(a + c)(b + d)] |  |
| BCPNN | IC=log_2_a(a+b+c+d)(a+c)(a+b) | IC_025_>0 |
|  | IC_025_=e^ln(IC)-1.96(1/a+1/b+1/c+1/d)^0.5^ |  |
| MGPS | EBGM=a(a+b+c+d)/((a+c)/(a+b)) | EBGM05 > 2, N > 0 |
|  | EBGM05=e^ln(EBGM)-1.64(1/a+1/b+1/c+1/d)^0.5^ |  |

a: the number of reports with suspect [adverse drug event](https://www.sciencedirect.com/topics/medicine-and-dentistry/adverse-drug-reaction) (ADE) of the suspect drug; b: the number of reports with all other ADEs of the suspect drug; c: the number of reports with the suspect ADE of all other drugs; d: the number of reports with all other ADEs of all other drugs; ROR: reporting odds ratio; CI: confidence interval; N: the number of co-occurrences; PRR: proportional reporting ratio; χ^2^: chi-squared; BCPNN: Bayesian confidence propagation neural network; IC: information component; IC025: the lower limit of the 95% two-sided CI of the IC; MGPS: multi-item gamma Poisson shrinker; EBGM: empirical Bayesian geometric mean; EBGM05: the lower 95% one-sided CI of EBGM.

**Supplementary Table S2.** Signal strength of reports of pertuzumab at the Preferred Terms (PTs) level from FAERS database.

^*^Emerging findings of pertuzumab associated AEs from FAERS database. ROR, reporting odds ratio; CI, confidence interval; PRR, proportional reporting ratio; χ, chi-squared; IC, information component; IC_025_, the lower limit of 95% CI of the IC; EBGM, empirical Bayesian geometric mean; EBGM_05_, the lower limit of 95% CI of EBGM.

| SOC | Preferred Terms (PTs) | Pertuzumab Cases Reporting PTs | ROR | PRR (χ^2^) | IC (IC_025_) | EBGM  (EBGM_05_) |
| --- | --- | --- | --- | --- | --- | --- |
| Blood and lymphatic system disorders | Myelosuppression | 305 | 86.09 (76.72-96.59) | 84.32 (16461.01) | 6.35 (5.66) | 81.77 (74.26) |
|  | Febrile neutropenia | 130 | 8.84 (7.44-10.51) | 8.77 (2406.09) | 3.13 (2.63) | 8.75 (7.57) |
|  | Neutropenia | 119 | 3.89 (3.24-4.65) | 3.86 (1411.93) | 1.95 (1.63) | 3.86 (3.32) |
|  | Agranulocytosis | 23 | 6.19 (4.11-9.32) | 6.18 (67.34) | 2.62 (1.74) | 6.17 (4.38) |
|  | Granulocytopenia | 8 | 6.38 (3.19-12.77) | 6.37 (8.24) | 2.67 (1.33) | 6.36 (3.56) |
|  | Haematotoxicity | 8 | 3.89 (1.95-7.79) | 3.89 (6.40) | 1.96 (0.98) | 3.89 (2.18) |
| Cardiac disorders | Cardiotoxicity | 70 | 35.04 (27.66-44.38) | 34.88 (837.72) | 5.11 (4.03) | 34.44 (28.26) |
|  | Tachycardia | 65 | 3.33 (2.61-4.25) | 3.32 (374.13) | 1.73 (1.36) | 3.32 (2.70) |
|  | Cardiac failure | 62 | 3.35 (2.61-4.30) | 3.34 (342.29) | 1.74 (1.35) | 3.34 (2.71) |
|  | Cardiac dysfunction | 27 | 34.95 (23.90-51.11) | 34.89 (124.62) | 5.11 (3.49) | 34.45 (25.07) |
|  | Left ventricular dysfunction | 27 | 17.27 (11.83-25.23) | 17.24 (117.22) | 4.10 (2.81) | 17.14 (12.48) |
|  | Cardiomyopathy | 22 | 6.98 (4.59-10.60) | 6.97 (64.34) | 2.80 (1.84) | 6.95 (4.90) |
|  | Pericardial effusion | 21 | 4.08 (2.66-6.26) | 4.07 (45.49) | 2.02 (1.32) | 4.07 (2.84) |
|  | Sinus tachycardia | 10 | 3.44 (1.85-6.40) | 3.44 (9.11) | 1.78 (0.96) | 3.43 (2.04) |
|  | Mitral valve incompetence | 9 | 4.38 (2.28-8.42) | 4.38 (8.74) | 2.13 (1.11) | 4.37 (2.53) |
|  | Ventricular hypokinesia | 8 | 12.39 (6.18-24.81) | 12.38 (9.80) | 3.62 (1.81) | 12.33 (6.89) |
| Gastrointestinal disorders | Diarrhoea | 833 | 5.62 (5.24-6.03) | 5.36 (84061.85) | 2.42 (2.26) | 5.35 (5.05) |
|  | Stomatitis | 73 | 4.95 (3.93-6.23) | 4.93 (614.35) | 2.30 (1.83) | 4.93 (4.06) |
|  | Colitis | 27 | 3.15 (2.16-4.60) | 3.15 (61.50) | 1.65 (1.13) | 3.14 (2.29) |
|  | Haemorrhoids | 15 | 3.09 (1.86-5.13) | 3.09 (18.64) | 1.63 (0.98) | 3.09 (2.02) |
|  | Enterocolitis | 11 | 8.58 (4.75-15.51) | 8.58 (17.11) | 3.10 (1.71) | 8.55 (5.21) |
|  | Intestinal perforation | 10 | 3.87 (2.08-7.20) | 3.87 (9.97) | 1.95 (1.05) | 3.87 (2.30) |
|  | Gastrointestinal toxicity | 6 | 5.89 (2.64-13.13) | 5.89 (4.50) | 2.56 (1.15) | 5.88 (3.01) |
| General disorders and administration site conditions | Pyrexia | 221 | 2.76 (2.42-3.16) | 2.74 (3584.59) | 1.45 (1.27) | 2.74 (2.45) |
|  | Disease progression | 196 | 7.45 (6.47-8.58) | 7.37 (5207.98) | 2.88 (2.50) | 7.35 (6.53) |
|  | Chills | 170 | 6.23 (5.35-7.24) | 6.17 (3682.21) | 2.62 (2.25) | 6.15 (5.42) |
|  | Mucosal inflammation | 73 | 12.39 (9.84-15.61) | 12.34 (815.66) | 3.62 (2.87) | 12.29 (10.13) |
|  | Chest discomfort | 69 | 2.95 (2.33-3.73) | 2.94 (375.97) | 1.55 (1.23) | 2.94 (2.41) |
|  | Hyperpyrexia | 14 | 16.56 (9.79-28.01) | 16.54 (31.35) | 4.04 (2.39) | 16.45 (10.59) |
|  | Temperature intolerance | 12 | 4.91 (2.79-8.65) | 4.91 (16.54) | 2.29 (1.30) | 4.90 (3.05) |
|  | Performance status decreased | 11 | 10.92 (6.04-19.75) | 10.92 (18.09) | 3.44 (1.90) | 10.88 (6.63) |
|  | Generalised oedema | 9 | 3.64 (1.90-7.01) | 3.64 (7.73) | 1.86 (0.97) | 3.64 (2.11) |
|  | Catheter site pain | 7 | 11.10 (5.28-23.33) | 11.10 (7.35) | 3.47 (1.65) | 11.05 (5.94) |
|  | Hyperthermia | 7 | 4.08 (1.94-8.56) | 4.08 (5.06) | 2.03 (0.96) | 4.07 (2.19) |
|  | Device related thrombosis | 5 | 19.48 (8.08-46.97) | 19.48 (4.08) | 4.27 (1.77) | 19.34 (9.26) |
|  | Mucosal disorder | 5 | 19.26 (7.99-46.42) | 19.25 (4.07) | 4.26 (1.77) | 19.12 (9.16) |
| Hepatobiliary disorders | Hepatic function abnormal | 44 | 5.73 (4.26-7.71) | 5.72 (238.93) | 2.51 (1.87) | 5.71 (4.45) |
|  | Hepatic lesion | 6 | 5.99 (2.69-13.34) | 5.98 (4.52) | 2.58 (1.16) | 5.97 (3.05) |
| Immune system disorders | Anaphylactic reaction | 50 | 4.30 (3.26-5.68) | 4.29 (266.70) | 2.10 (1.59) | 4.29 (3.40) |
|  | Anaphylactic shock | 28 | 5.40 (3.72-7.82) | 5.39 (94.26) | 2.43 (1.68) | 5.38 (3.94) |
|  | Sepsis | 65 | 2.50 (1.96-3.19) | 2.49 (275.03) | 1.32 (1.03) | 2.49 (2.03) |
|  | Cellulitis | 48 | 3.83 (2.89-5.09) | 3.82 (227.82) | 1.93 (1.46) | 3.82 (3.01) |
|  | Device related infection | 29 | 6.67 (4.63-9.61) | 6.66 (110.08) | 2.73 (1.90) | 6.65 (4.90) |
|  | Neutropenic sepsis | 20 | 11.75 (7.57-18.23) | 11.73 (60.65) | 3.55 (2.29) | 11.69 (8.09) |
|  | Skin infection | 14 | 5.16 (3.05-8.71) | 5.15 (23.07) | 2.36 (1.40) | 5.14 (3.32) |
|  | Erysipelas | 13 | 10.51 (6.10-18.13) | 10.51 (25.07) | 3.39 (1.96) | 10.47 (6.64) |
|  | Gastroenteritis | 12 | 3.67 (2.08-6.46) | 3.67 (13.80) | 1.87 (1.06) | 3.66 (2.28) |
|  | Rash pustular | 11 | 6.29 (3.48-11.37) | 6.29 (15.50) | 2.65 (1.47) | 6.27 (3.82) |
|  | Paronychia | 10 | 9.81 (5.27-18.26) | 9.80 (14.61) | 3.29 (1.77) | 9.77 (5.81) |
|  | Wound infection | 10 | 4.55 (2.44-8.46) | 4.54 (11.02) | 2.18 (1.17) | 4.54 (2.70) |
|  | Subcutaneous abscess | 9 | 7.52 (3.91-14.46) | 7.51 (11.03) | 2.91 (1.51) | 7.49 (4.33) |
|  | Vascular device infection | 8 | 8.22 (4.11-16.47) | 8.22 (8.95) | 3.04 (1.52) | 8.20 (4.59) |
|  | Enterocolitis infectious | 6 | 29.56 (13.22-66.11) | 29.55 (6.09) | 4.87 (2.18) | 29.24 (14.91) |
| Injury, poisoning and procedural complications | Infusion related reaction | 113 | 7.75 (6.44-9.33) | 7.70 (1753.22) | 2.94 (2.44) | 7.68 (6.58) |
|  | Radiation skin injury | 6 | 22.37 (10.01-49.97) | 22.36 (5.95) | 4.47 (2.00) | 22.18 (11.32) |
|  | Radiation necrosis | 5 | 47.26 (19.52-114.45) | 47.24 (4.34) | 5.54 (2.29) | 46.44 (22.16) |
| Investigations | Ejection fraction decreased | 109 | 31.58 (26.13-38.17) | 31.35 (2017.95) | 4.95 (4.10) | 31.00 (26.45) |
|  | White blood cell count decreased | 68 | 2.57 (2.03-3.26) | 2.56 (312.39) | 1.36 (1.07) | 2.56 (2.10) |
|  | Oxygen saturation decreased | 52 | 4.12 (3.14-5.42) | 4.11 (280.91) | 2.04 (1.55) | 4.11 (3.27) |
|  | Neutrophil count decreased | 44 | 4.79 (3.56-6.44) | 4.78 (219.40) | 2.25 (1.68) | 4.77 (3.72) |
|  | SARS-CoV-2 test positive | 15 | 3.98 (2.40-6.60) | 3.97 (22.83) | 1.99 (1.20) | 3.97 (2.60) |
|  | Blood magnesium decreased | 13 | 6.42 (3.72-11.06) | 6.41 (21.82) | 2.68 (1.55) | 6.40 (4.06) |
|  | Red cell distribution width increased | 12 | 11.85 (6.72-20.89) | 11.84 (21.87) | 3.56 (2.02) | 11.79 (7.33) |
|  | Blood creatine increased | 9 | 10.22 (5.31-19.67) | 10.21 (11.94) | 3.35 (1.74) | 10.18 (5.88) |
|  | Blood pressure diastolic decreased | 9 | 4.96 (2.58-9.53) | 4.95 (9.35) | 2.31 (1.20) | 4.95 (2.86) |
|  | Pulse absent | 8 | 9.02 (4.51-18.06) | 9.02 (9.17) | 3.17 (1.58) | 8.99 (5.03) |
|  | Mean platelet volume decreased | 7 | 36.31 (17.22-76.55) | 36.29 (8.40) | 5.16 (2.45) | 35.81 (19.19) |
|  | Granulocyte count decreased | 7 | 30.95 (14.69-65.21) | 30.93 (8.31) | 4.93 (2.34) | 30.59 (16.40) |
|  | Tumour marker increased | 7 | 5.99 (2.85-12.59) | 5.99 (6.16) | 2.58 (1.23) | 5.98 (3.21) |
|  | Carbohydrate antigen 15-3 increased | 6 | 36.77 (16.42-82.31) | 36.75 (6.17) | 5.18 (2.31) | 36.27 (18.48) |
|  | Echocardiogram abnormal | 5 | 18.64 (7.73-44.93) | 18.63 (4.06) | 4.21 (1.75) | 18.51 (8.87) |
| Metabolism and nutrition disorders | Dehydration | 127 | 4.21 (3.54-5.02) | 4.19 (1696.31) | 2.06 (1.73) | 4.18 (3.61) |
|  | Hypokalaemia | 53 | 5.20 (3.97-6.81) | 5.19 (331.83) | 2.37 (1.81) | 5.18 (4.13) |
|  | Tumour lysis syndrome | 13 | 6.52 (3.78-11.23) | 6.51 (21.94) | 2.70 (1.57) | 6.50 (4.12) |
|  | Hypomagnesaemia | 12 | 3.93 (2.23-6.93) | 3.93 (14.51) | 1.97 (1.12) | 3.93 (2.44) |
| Neoplasms benign, malignant and unspecified (incl cysts and polyps) | Metastases to central nervous system | 88 | 30.93 (25.05-38.18) | 30.75 (1313.53) | 4.93 (3.99) | 30.41 (25.49) |
|  | Breast cancer metastatic | 25 | 12.53 (8.45-18.56) | 12.51 (95.87) | 3.64 (2.46) | 12.45 (8.96) |
|  | Metastases to bone | 24 | 6.42 (4.30-9.58) | 6.41 (74.34) | 2.68 (1.79) | 6.39 (4.57) |
|  | Metastases to liver | 23 | 5.49 (3.64-8.26) | 5.48 (64.07) | 2.45 (1.63) | 5.47 (3.88) |
|  | Metastasis | 22 | 13.35 (8.78-20.31) | 13.34 (75.04) | 3.73 (2.45) | 13.27 (9.35) |
|  | Breast cancer recurrent | 20 | 29.63 (19.06-46.05) | 29.59 (67.66) | 4.87 (3.13) | 29.28 (20.24) |
|  | Metastases to lung | 18 | 6.65 (4.19-10.56) | 6.64 (42.37) | 2.73 (1.72) | 6.63 (4.50) |
|  | Metastases to lymph nodes | 13 | 8.86 (5.14-15.28) | 8.86 (24.10) | 3.14 (1.82) | 8.83 (5.60) |
|  | Metastatic neoplasm | 10 | 10.97 (5.89-20.42) | 10.97 (14.97) | 3.45 (1.85) | 10.92 (6.50) |
|  | Brain neoplasm | 10 | 4.50 (2.42-8.36) | 4.49 (10.95) | 2.17 (1.16) | 4.49 (2.67) |
|  | Recurrent cancer | 8 | 8.40 (4.20-16.82) | 8.40 (9.00) | 3.07 (1.53) | 8.38 (4.69) |
|  | Metastases to skin | 7 | 28.53 (13.55-60.10) | 28.52 (8.27) | 4.82 (2.29) | 28.23 (15.14) |
|  | Breast neoplasm | 5 | 19.89 (8.25-47.95) | 19.89 (4.09) | 4.30 (1.79) | 19.75 (9.46) |
| Nervous system disorders | Neuropathy peripheral | 128 | 5.67 (4.77-6.75) | 5.63 (2010.91) | 2.49 (2.09) | 5.62 (4.86) |
|  | Polyneuropathy | 24 | 9.06 (6.07-13.53) | 9.05 (82.58) | 3.17 (2.12) | 9.02 (6.45) |
|  | Intracranial pressure increased | 6 | 4.87 (2.18-10.84) | 4.86 (4.12) | 2.28 (1.02) | 4.86 (2.48) |
| Reproductive system and breast disorders | Breast pain | 13 | 4.94 (2.87-8.51) | 4.93 (19.47) | 2.30 (1.33) | 4.93 (3.12) |
|  | Breast disorder | 6 | 11.90 (5.34-26.54) | 11.90 (5.47) | 3.57 (1.60) | 11.85 (6.06) |
| Respiratory, thoracic and mediastinal disorders | Interstitial lung disease | 136 | 12.78 (10.79-15.13) | 12.67 (2844.95) | 3.66 (3.09) | 12.61 (10.95) |
|  | Pneumonitis | 52 | 8.54 (6.50-11.22) | 8.52 (381.76) | 3.09 (2.35) | 8.49 (6.76) |
|  | Pleural effusion | 50 | 3.60 (2.73-4.75) | 3.59 (235.99) | 1.84 (1.40) | 3.59 (2.84) |
|  | Bronchospasm | 23 | 7.36 (4.88-11.08) | 7.35 (71.54) | 2.87 (1.91) | 7.33 (5.20) |
|  | Pulmonary hypertension | 16 | 3.23 (1.98-5.28) | 3.23 (22.11) | 1.69 (1.03) | 3.23 (2.14) |
|  | Pulmonary mass | 14 | 3.50 (2.07-5.92) | 3.50 (18.13) | 1.81 (1.07) | 3.50 (2.26) |
|  | Lung infiltration | 13 | 7.85 (4.55-13.54) | 7.85 (23.31) | 2.97 (1.72) | 7.83 (4.96) |
|  | Nasal ulcer | 6 | 14.95 (6.70-33.35) | 14.94 (5.68) | 3.89 (1.75) | 14.86 (7.59) |
| Skin and subcutaneous tissue disorders | Rash | 294 | 2.91 (2.59-3.27) | 2.87 (6699.55) | 1.52 (1.35) | 2.87 (2.61) |
|  | Palmar-plantar erythrodysaesthesia syndrome | 41 | 7.30 (5.37-9.93) | 7.29 (226.78) | 2.86 (2.11) | 7.27 (5.62) |
|  | Dermatitis acneiform | 20 | 14.86 (9.57-23.06) | 14.84 (63.04) | 3.88 (2.50) | 14.76 (10.22) |
|  | Skin reaction | 19 | 5.49 (3.50-8.62) | 5.49 (43.74) | 2.45 (1.56) | 5.48 (3.76) |
|  | Nail disorder | 17 | 9.09 (5.64-14.64) | 9.08 (41.47) | 3.18 (1.97) | 9.05 (6.08) |
|  | Skin toxicity | 13 | 10.93 (6.34-18.85) | 10.92 (25.27) | 3.44 (2.00) | 10.88 (6.90) |
|  | Onychomadesis | 21 | 26.77 (17.41-41.16) | 26.74 (74.04) | 4.73 (3.07) | 26.48 (18.48) |
|  | Onychoclasis | 11 | 6.14 (3.40-11.10) | 6.14 (15.36) | 2.62 (1.45) | 6.13 (3.73) |
|  | Nail discolouration | 10 | 10.13 (5.44-18.85) | 10.12 (14.72) | 3.33 (1.79) | 10.09 (6.00) |
| Surgical and medical procedures | Mastectomy | 13 | 37.81 (21.86-65.38) | 37.78 (29.02) | 5.22 (3.02) | 37.26 (23.56) |
|  | Breast operation | 7 | 50.16 (23.74-105.98) | 50.14 (8.53) | 5.62 (2.66) | 49.23 (26.33) |
| Vascular disorders | Cyanosis | 15 | 4.85 (2.92-8.06) | 4.85 (25.70) | 2.28 (1.37) | 4.84 (3.17) |
|  | Lymphoedema | 14 | 8.39 (4.96-14.18) | 8.38 (27.55) | 3.06 (1.81) | 8.36 (5.39) |
|  | Circulatory collapse | 13 | 3.49 (2.02-6.01) | 3.48 (15.57) | 1.80 (1.04) | 3.48 (2.21) |
